# What are the determinants of vegetable intake among adolescents from socioeconomically disadvantaged urban areas? A systematic review of qualitative studies

**DOI:** 10.1186/s12966-022-01396-9

**Published:** 2022-12-26

**Authors:** Silvia Bel-Serrat, Antje von der Schulenburg, Maria Marques-Previ, Amy Mullee, Celine M Murrin

**Affiliations:** 1 National Nutrition Surveillance Centre, School of Public Health, Physiotherapy and Sports Science, University College Dublin, Woodview House, Belfield, Dublin, Ireland; 2 Department of Health and Nutritional Sciences, Atlantic Technological University, Sligo, Ireland

**Keywords:** Vegetable intake, Determinants, Adolescents, Socioeconomically disadvantaged, Qualitative methods

## Abstract

**Background:**

Evidence available on the determinants of vegetable intake in young populations is inconsistent. Vegetable intake is particularly low in adolescents from less-affluent backgrounds, yet no systematic review of qualitative studies investigating determinants for vegetable intake specifically has been conducted to date in this group. This systematic review aimed to identify determinants of vegetable intake in adolescents from socioeconomically disadvantaged urban areas located in very high-income countries reported in qualitative studies.

**Methods:**

Five electronic databases (PubMed, Web of Science, CINAHL, PsycINFO and ERIC) were searched until August 2022. The search strategy used combinations of synonyms for vegetable intake, adolescents, and qualitative methodologies. Main inclusion criteria were studies exploring views and experiences of motivators and barriers to vegetable intake in a sample of adolescents aged 12–18 years from socioeconomically disadvantaged urban areas in very high income countries. Study quality assessment was conducted using criteria established in a previous review.

**Results:**

Sixteen studies were included out of the 984 screened citations and 63 full texts. The synthesis of findings identified the following determinants of vegetable intake: sensory attributes of vegetables; psychosocial factors (nutrition knowledge, preferences/liking, self-efficacy, motivation); lifestyle factors (cost/price, time, convenience); fast food properties (taste, cost, satiety); home environment and parental influence; friends’ influence; school food environment, nutrition education and teachers’ support; and availability and accessibility of vegetables in the community and community nutrition practices. Studies attained between 18 and 49 out of 61 quality points, with eleven of 16 studies reaching ≥ 40 points. One main reason for lower scores was lack of data validation.

**Conclusion:**

Multiple determinants of vegetable intake were identified complementing those investigated in quantitative studies. Future large scale quantitative studies should attempt to examine the relative importance of these determinants in order to guide the development of successful interventions in adolescents from socioeconomically disadvantaged backgrounds.

**Supplementary Information:**

The online version contains supplementary material available at 10.1186/s12966-022-01396-9.

## Introduction

One of the four modifiable behavioral risk factors that increase the risk of non-communicable diseases, such as cardiovascular diseases, cancers, chronic respiratory diseases and diabetes, is unhealthy diets [1]. In order to attain a healthy diet, the World Health Organization (WHO) recommends an intake of a minimum of 400 grams/day of fruit and vegetables [2]. However, international surveys conducted among adolescents aged 11–15 years showed that only 48% eat fruit and vegetables on a daily basis and 38% eat vegetables daily, with consumption generally declining with advancing age [3]. Further, intake was lower in adolescents from less affluent backgrounds in most developed countries, highlighting the presence of food-related social inequalities [3]. Therefore, urgent action is needed to promote fruit and vegetable intake among young people, particularly among those from lower socioeconomic backgrounds, to tackle these inequalities in food consumption. However, in order to develop effective policies and interventions, it is important to identify determinants of fruit and vegetable intake in this population, yet the evidence specific to adolescents from socioeconomically disadvantaged backgrounds remains limited. In addition to socioeconomic inequalities, existing evidence suggests a notable difference in the availability of healthy foods and prevalence of related health conditions such as obesity between urban and rural settings [4–6]. For that reason, policies and intervention programs may need to be tailored to both the socioeconomic level and the place of residence of their target population.

There is a lack of information on the determinants specific to vegetable intake among adolescents from socioeconomically disadvantaged backgrounds. In their systematic review of quantitative studies, Di Noia and Byrd-Bredbenner identified maternal fruit and vegetable intake and own fruit and vegetable preferences to be consistently associated with the intake of fruit and vegetables in youth from low-income backgrounds aged < 20 years [7]. However, this review was not specific to adolescents and vegetables in isolation. A systematic review focusing, on qualitative studies investigating the determinants of fruit and vegetable intake among children and adolescents was conducted by Krølner et al. [8]. Although relevant, the review focused on adolescents from all socioeconomic backgrounds and, again, the determinants were not examined individually for fruit and vegetables.

It is crucial to distinguish between the determinants of intake of fruit and vegetables. Epidemiological and intervention studies have largely focused on fruit and vegetables in combination. This may be due to the fact that they share certain health benefits as a result of their constituent bioactive compounds, such as, vitamins, minerals, antioxidants, carotenoids and flavonoids [9–11]. However, this review focuses on determinants of vegetable intake independent of the determinants of fruit intake for a number of reasons. Firstly, studies that investigated fruits and vegetables separately found differential effects on health outcomes in adults [12–14]. This may be due to their different nutritional profile regarding sugars, protein, and fiber [11] and the type and concentration of bioactive compounds. Furthermore, vegetables usually need to be processed prior to their consumption, which affects the bioavailability of these bioactive compounds in different ways [11, 15–21]. Secondly, fruits and vegetables taste differently, have different textures and are consumed in different manners. While fruits are mostly sweet and are usually consumed raw as a snack, drink or a dessert, vegetables can taste bitter, often need to be cooked and are frequently consumed as part of a meal [22–24]. These different consumption patterns between fruits and vegetables may suggest that their intakes are determined by different factors [9]. This may partially explain why school-based interventions aiming to improve fruit and vegetable intake in children seem to moderately improve fruit intake, while they have a limited impact on vegetable intake [25]. For that reason, fruit and vegetables and their determinants of intake need to be investigated separately and targeted independently by intake promotion policies and interventions.

Understanding the context in which health behaviours occur is key to developing successful public health programs [26]. The socio-ecological model (SEM) of health uses a five-level approach that takes into account the interplay between individual, interpersonal (family and friends), organizational (school), community and public policy factors [27, 28] and illustrates how factors at one level influence factors at another level [28].Therefore, effective prevention strategies need to promote changes in the physical and the social environment rather than just focusing on individual behavior change [27].

Quantitative and qualitative studies should be used in parallel to provide more comprehensive understanding of the contexts for health behaviors. We previously conducted a systematic review on the determinants of vegetable intake in adolescents from socioeconomically disadvantaged urban areas examined through quantitative studies. Nutrition knowledge was the only determinant that was consistently investigated in several independent studies; however, it emerged that it was not related to vegetable intake among adolescents from socioeconomically disadvantaged backgrounds [29]. Other determinants evaluated included self-efficacy, subjective norms, or preferences, but there were not enough studies to examine the consistency of the evidence for these determinants and a conclusion could not be reached. While quantitative research provides information about general patterns of behavior at the population level, among other aspects, it cannot provide a rich understanding around attitudes, perceptions or behaviors of a specific topic [30]. Qualitative research, on the other hand, can offer an understanding of why people do what they do [30]. Therefore, qualitative studies complement quantitative findings, as participants are given the opportunity to provide unique answers on factors that were not initially contemplated and that otherwise would have not been investigated, generating a more thorough understanding of that phenomenon [8, 31, 32]. Hence, this systematic review aims to explore the views and experiences of adolescents from socioeconomically disadvantaged backgrounds in urban areas on the determinants of healthy eating, particularly of vegetable intake, collected with qualitative methodologies. Results will be used to inform the development of an intervention study to promote vegetable intake among adolescents aged 13–15 years from socioeconomically disadvantaged urban areas in a very high-income country. To our knowledge, this is the first systematic review of this kind.

## Methods

We conducted a systematic review in line with the Preferred Reporting Items for Systematic Reviews and Meta-Analyses guidelines [33] and registered with the International Prospective Register of Systematic Reviews (PROSPERO) with registration ID CRD42020188110. A review protocol was developed to define the methods of the systematic review.

The age range selected for adolescents in this review was 12–18 years old based on the age ranges commonly used in the stages of the school system, i.e., pre-school (< 6 years), primary school (6–12 years) and secondary school (12–18 years). Therefore, the target population of this systematic review was adolescents aged 12–18 years. The sample was considered as socioeconomically disadvantaged when either the study setting or the study population were described as such in the manuscript.

### Search strategy

Five electronic bibliographic databases (PubMed, Web of Science, CINAHL, ERIC and PsycINFO) were searched from inception until October 8, 2020 to identify relevant studies. The list of studies was updated with a second search on August 25, 2022. We applied the same electronic search strategy in all databases by combining key search terms for the following 3 categories: vegetable, population of interest (e.g., adolescents, youth), and qualitative methods and methodologies (e.g., anthropology, ethnography, qualitative, focus group, interview). No specific keywords were used for socioeconomic status to retrieve as many studies as possible. The search carried out in PubMed is provided as additional information (Additional file 1). Additional studies were identified by means of manual searches of reference lists of previously published reviews and of included papers.

### Inclusion and exclusion criteria

The purpose of this review is to inform the development of an intervention program to promote vegetable intake in a population of adolescents from socioeconomically disadvantaged urban areas in a very high-income country. Hence, studies were included if: (1) the sample comprised individuals from a socioeconomically disadvantaged background (or with the majority of adolescents from a disadvantaged background or comparing adolescents from a non-disadvantaged vs. disadvantaged background) aged between 12 and 18 years (or with a mean age between 12 and 18 years), (2) investigated at least one determinant of vegetable intake, either as the primary focus or as part of healthy eating (diet, nutrition or food) where information specifically related to vegetables could be identified, or (3) explored views and experiences of motivators and barriers to vegetable intake, and (4) were conducted in urban settings (described as such by the study researchers, or the study was conducted in a setting described as urban and/or in a large well-known urban area, e.g., Baltimore City, Boston, etc., or there was a majority of study participants from urban areas) of very high income countries according to the Human Development Index (HDI) 2019 from the United Nations Development Programme (HDI ≥ 0.800) [34], (5) were published in English-, French-, Spanish-, Portuguese- or Catalan languages, (6) applied qualitative research methods, and (7) were published in peer-reviewed journals. Studies that reported findings from parents and/or other adults, e.g., schoolteachers, on the determinants of adolescents’ vegetable intake were also included.

Studies were excluded if they: (1) had a quantitative methodology or methodological aims, or (2) were reviews, meta-analyses, or (3) were intervention studies without qualitative methods or with qualitative data collection exclusively applied to assess the feasibility of the intervention, (4) were not conducted in healthy populations, (5) were conducted in settings explicitly described as rural or with a majority of rural participants, (6) focused exclusively on participants with overweight and obesity, and (7) focused exclusively on sociodemographic determinants such as sex, age, socioeconomic position, race/ethnicity or urbanization.

### Study selection and data extraction

Two reviewers (SBS and AM) independently screened titles and abstracts of 10% of all the retrieved articles against the study selection criteria. Any discrepancies were resolved by consensus and then, one reviewer (SBS) screened the remaining 90% of the titles and abstracts and excluded irrelevant records. Of all records included based on title/abstract, the full texts were assessed to make conclusions about inclusion in the review. Again, 10% of full-text papers that either met the eligibility criteria or had insufficient information in the abstract to determine eligibility were independently reviewed by two reviewers (SBS and AM) and disagreements were discussed until an agreement was reached. One reviewer (SBS) reviewed the full text of the remaining papers and determined the final pool of articles included in the review.

Data extraction was performed by two independent reviewers (SBS and AVDS) using an Excel spreadsheet to collect key data from each study. Information was extracted on first author and year of publication, phenomenon of interest, sampling of participants, adolescent’s characteristics (sex, age, race/ethnicity, setting and country), data collection methods and number of focus groups and/or interviews, theoretical framework, analytical method, and main topics related to vegetable intake. The extracted items were drawn from prior reviews in order to allow comparisons among studies [8].

### Data synthesis

Data from all studies were synthesized using the approach followed by Krølner et al. [8]. All key findings related to vegetable intake as well as their respective illustrative quotations were extracted by entering the data into a table. Once extracted, another table was populated summarizing the findings from all the studies to facilitate their comparison. This comparison was made systematically to identify similarities and differences. This involved going back and forth between the original papers, their data extractions and the summary table with the findings. Then, findings were coded and categorized into themes as follows: (1) findings that were similar or represented varying aspects of the same theme were grouped under that theme, and (2) findings that were different were separated and renamed into other themes [8, 35, 36]. We extracted each finding and quotation together with their country of origin to point out any existing country-specific differences in the results [8]. An example of the coding and grouping of the findings into themes in provided in Additional file 2.

### Study quality assessment

Two independent reviewers (SBS and AVDS) carried out a systematic assessment of the methodological quality of each paper. We applied the list of quality criteria for papers with a qualitative methodology described by Krølner et al. [8] including: (1) methodological aspects explicitly and clearly explained in the paper (sampling procedure, sample characteristics, ethical concerns, data collection and data analyses), (2) internal validity (validity and pilot testing of the methods applied, triangulation of researchers, methods and sources, etc.), (3) external validity (transferability of findings), and (4) pragmatic validity (how study findings could inform future research and practice). The overall quality of the papers was assessed with a count of the total of criteria met [8]. Disagreements in assessments were resolved through discussions, therefore, there was no need to involve a third reviewer. The study quality assessment was used to examine the strength of scientific evidence but did not determine the inclusion of the studies in the review.

## Results

### Study selection

**Fig. 1:**
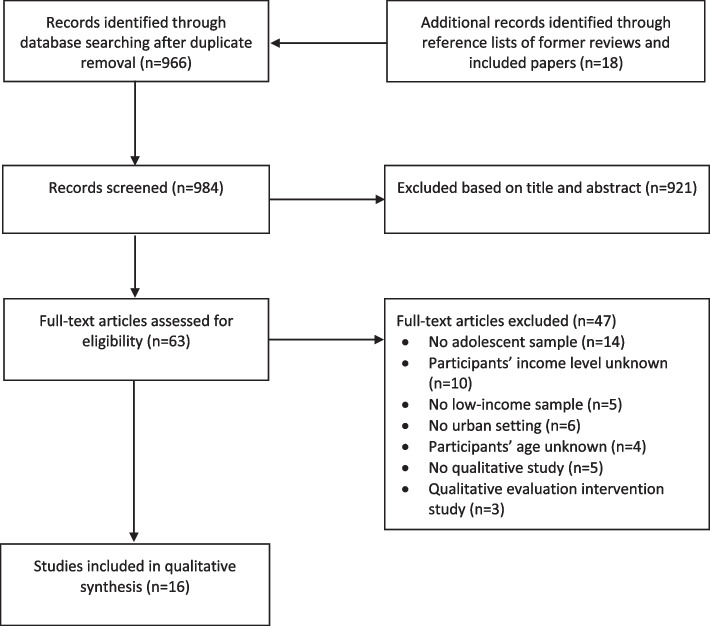
Flow chart of the study selection process

A total of 984 records, excluding duplicates, were retrieved. Of these, 966 records were obtained from database searches and 18 via reference lists of existing reviews and included papers. After screening of titles and abstracts, 63 articles remained for full-text screening. Among these, 16 studies met the inclusion criteria and were included in this review (Fig. 1). All the studies included in this review were published in English.

### Study characteristics

Table 1 summarizes the characteristics of the 16 included studies that met the inclusion criteria. Studies were published between 1999 [37] and 2019 [38]. Seven studies were from the late 2000’s [39–45]. Most of the studies (*n* = 8) were published between 2013 and 2019 [38, 46–52]. Twelve studies were conducted in the United States (US) [37, 39–47, 50, 51], two in Europe [48, 49], one in Australia [52] and another one in New Zealand [38]. Eight studies included a range of ethnic groups [37–40, 42, 44, 50, 51], three studies exclusively focused on African American populations [41, 46, 47], one study [43] targeted Asian American people including Chinese, Vietnamese and Hmong participants, and one study [45] exclusively focused on Hmong (Southeast Asian origin) participants. Three manuscripts [48, 49, 52] did not provide details about race/ethnicity. All studies targeted mixed-gender samples, except four studies [40, 43, 46, 48] with no information on participants’ gender.

**Table 1:** Summary of study characteristics

Author, publication year	Phenomenon of interest (outcome)	Sampling of participants	Sample characteristics (sex, age, race/ethnicity, setting, country)	Data collection methods, number of focus groups/interviews	Theoretical framework	Analytical method/approach
Campbell, 2009 [39]	Dietary choices	School-based: 12 adolescents	Sex: mixed Age: 14-16 years (9th-10th grades) Race/ethnicity: 8 Hispanic, 2 bi-racial African American Hispanic and Caucasian Hispanic, 1 Eurasian and 1 African American Setting: public high school Country: US	FG discussions: one group interviewed twice, during lunch and immediately after	Developmental psychology by Piaget and Erikson	No detailed description of analytical procedures Content analyses
Chatterjee, 2016 [46]	School food	School-based (1 school): 32 students, 10 parents, 14 faculty/staff, 3 school leadership roles.	Sex: no information Age: 13-19 years (9th-12th graders) Race/ethnicity: 98% "color" Setting: public high school Country: US	FG discussions: 1 9th grade students FG, 1 10th grade students FG, 1 11th grade students FG, 1 12th grade students FG, 2 parent FGs, 1 teacher/staff FG. Interviews: 3 school leadership roles	No information	Immersion-crystallization method
Christiansen, 2013 [47]	Eating habits	Community-based: 20 adolescents	Sex: mixed Age: 10-16 years Race/ethnicity: African American Setting: recreation centers Country: US	2 adolescents FG discussions, 18 adolescents interviews plus direct observation in stores	Ecological model from Bronfenbrenner and social cognitive theory	Significant themes identified before coding. Themes were subsequently categorized, and a codebook was developed for use during the coding process
Cullen, 2007 [40]	School food	School-based (6 schools): schoolchildren, schoolstaff (number of participants not provided) and 7 district school food administrators	Sex: no info Age: 11-14 years Race/ethnicity: African Americans, Hispanics, White, Native American, other Setting: middle schools Country: US	11 FGs with schoolchildren and school staff. 7 interviews with district school food administrators	No information	No detailed description of analytical procedures
Dalma, 2016 [48]	Healthy eating	School-based (14 schools): 98 schoolchildren (28 8-10 years schoolchildren, 31 10-12 years schoolchildren, 39 junior high school students) and 44 parents (22 parents elementary school students, 22 parents junior high school students)	Sex: no info Age: 9-15 years (3rd, 4th, 5th and 6th graders, junior high school) Race/ethnicity: no info Setting: elementary and secondary public schools Country: Greece	20 FGs with school children and parents: 4 FGs 8-10 years schoolchildren, 4 FGs 10-12 years schoolchildren, 6 FGs junior high school students, 3 FGs parents elementary school students, 3 FGs parents junior high school students.	No information	Krueger’s ‘long table approach’ method
Davison, 2015 [49]	Healthy eating	Community-based: 14 young people attending "Not in education, employment or training" youth support services, 7 service providers	Sex: mixed Age: 16-20 years Race/ethnicity: no information Setting: youth support services Country: Northern Ireland, UK	3 FGs with young people. 6 interviews and one-paired interview with service providers (6 females and one male).	Social cognitive theory	Thematic content analyses
Dodson, 2009 [41]	Food consumption behavior	Community-based (3 centers): samples size not clearly reported for adolescents, 7 adults (1 teacher, 1 parent, 1 storeowner, 4 community center administrators)	Sex: mixed Age: 12-17 years Race/ethnicity: African American Setting: community organizations Country: US	15 interviews with 9 adolescents (3 boys and 6 girls), 1 FG with 5 girls and a paired interview with boys, 5 girls took pictures, 1 FG with 3 girls and paired interview with 2 boys, 7 interviews with adults plus observation in stores	Social cognitive theory	Data obtained through qualitative methods with adolescents were triangulated by conducting participant observation and interviews with adults in the community. Interviews data were analyzed using content analysis and coding techniques
Gerritsen, 2019 [38]	Fruit and vegetable intake	Community-based: 17 students, parents,teachers, community leaders, local retailers, and health promoters	Sex: mixed Age: 16-25 years Race/ethnicity: Maori, Pacific, Asian and New Zealand European Setting: neighborhood community Country: New Zealand	Three group model building workshops with all participants together	No information	Structured system dynamics method to create a causal loop diagram
Goh, 2009 [42]	Healthy eating (and physical activity)	School- based (2 schools): 119 adolescents, 63 parents, and 28 community stakeholders	Sex: mixed Age: mean 12 years (7th-8th graders) Race/ethnicity: 58% Latino Setting: middle school Country: US	FG discussions: 6 male teenagers FGs, 8 female teenagers FGs, 8 parent FGs. 28 interviews with community stakeholders	No information	No information
Greer, 2018 [50]	Local produce consumption	School-based (3 schools): 53 students	Sex: mixed Age: no information Race/ethnicity: non-Hispanic black, Hispanic, non-Hispanic white and Asian Setting: public high schools Country: US	6 students FGs (2 FGs per school)	Social cognitive theory	No information
Harrison, 2005 [43]	Dietary habits, attitudes and knowledge	Community-based: 236 participants (15 key informants, 116 parents, and 105 youth participants)	Sex: no info Age: 11-14 years Race/ethnicity: Asian American (Chinese, Vietnamese, and Hmong) Setting: community-based organizations Country: US	24 youth and parents FGs 15 interviews with key informants	No information	Simple thematic content analysis was used to summarize the data by ethnic group and across groups
Kubik, 2005 [44]	Dietary practice (and physical activity)	School-based (7 alternate high schools): 70 students	Sex: mixed Age: 9th-12th graders Race/ethnicity: non-Caucasian origin (American Indian, African American, Hispanic and Asian) Setting: alternative high school Country: US	7 students FGs	Social cognitive theory and ecologic theory are included in the discussion	Systematic three-step process as described by Miles and Huberman (1994)
Neumark-Sztainer, 1999 [37]	Food choices and eating behaviors	School-based (2 schools): 141 students	Sex: mixed Age: mean age 12.6 years (12-14 years) and mean age 16 years (15-19 years) Race/ethnicity: white, Asian American, African American, multiracial, Hispanic and Native American Setting: high school Country: US	21 students FGs: 7 7th grade female students FGs, 5 7th grade male students FGs, 4 10th grade female students FGs and 5 10th grade male students FGs	Social cognitive theory is included in the discussion	Systematic analytical approach using the constant comparative method of grounded theory
Payan, 2017 [51]	Healthy eating and school food	School-based (3 schools): 64 students	Sex: mixed Age: mean age 16.3 years (14-19 years) Race/ethnicity: Hispanic/Latino (59.4%) and African American (32.8%) Setting: high school Country: US	8 students FGs	Self-determination theory is included in the discussion	Grounded theory
Pham, 2007 [45]	Dietary practices around fruit and vegetable intake (and physical activity)	Community-based: 40 youth participants, 44 parents, and 5 key informants	Sex: mixed Age: 11-14 years Race/ethnicity: Hmong (Southeast Asian origin) Setting: community-based organizations Country: US	8 FGs with youth and parents 5 key informants interviews	No information	No detailed description of analytical procedures. Data were coded and content analyzed
Stephens, 2015 [52]	Fruit and vegetable intake	School-based (3 schools): 22 adolescents	Sex: mixed Age: 12-15 years (7th and 8th graders) Race/ethnicity: no information Setting: secondary schools Country: Australia	22 telephone interviews with adolescents	Social ecological theory	Grounded theory and inductive thematic analysis

*US* United States, *FG* focus groups

Nine studies [38, 40–43, 45, 46, 48, 49] used source triangulation by interviewing parents, school staff, key informants from school, including school leadership roles and district school food administrators, and/or from the community, e.g., youth service providers, local retailers, community center administrators, community leaders or health promoters, in addition to adolescents. The majority of the participants were recruited through either high schools [37, 39, 46, 48, 50–52] or middle schools [40, 42]. Four studies used community organizations [38, 41, 43, 45]. Three studies focused on adolescents attending alternative schools [44], recreation centers [47] or youth support services [49]. Half of the studies combined focus groups and interviews to collect data from participants [40–43, 45–47, 49], six studies exclusively used focus groups [37, 39, 44, 48, 50, 51], one study collected data through phone interviews [52] and another study used model building workshops [38]. Two studies also gathered data through direct observation [41, 47].

Six studies were grounded in theoretical frameworks [39, 41, 47, 49, 50, 52]. Among these, three studies [41, 49, 50] exclusively applied the Social Cognitive Theory (SCT) and one study [47] combined the SCT with the Social Ecological Model from Bronfenbrenner. Three studies included the SCT [37, 44], the ecologic theory [44], and the self-determination theory [51] in their discussion sections; however, it is unclear if these theories were applied in any aspect of the study. Seven studies did not provide any information about the use of theoretical frameworks in their studies [38, 40, 42, 43, 45, 46, 48]

### Synthesis of findings

A total of six main themes were identified (Table 2): 1) vegetables characteristics, 2) personal factors, 3) fast foods versus vegetables, 4) family, home and friends, 5) school, and 6) community. Each theme is described in more detail below. Themes were organized into five levels according to the SEM [28]: individual, interpersonal, organizational, community and public policy. There were no themes identified that aligned with the policy level.

**Table 2:** Themes from analyses and supporting excerpts/quotes

**Themes/sub-themes**	**Supporting excerpts & quotes**	**Contributing studies**
Vegetables characteristics		
Sensory attributes	“Cut [up] fresh, not old fruits or vegetables. If it smells good, then it will make you want to eat more.” [45]	[37, 45, 50]
Personal factors		
Cognitive factors	Like parents, many children reported that the foods they disliked were legumes, cooked vegetables and fish. [48]	[37, 39, 41–45, 47–49, 51]
	Participants’ knowledge of food groups was minimal. Several did not distinguish between fruits and vegetables. [39]	
	Family histories of obesity, diabetes, and heart disease were listed as factors motivating adolescents to consume food identified as healthy, such as fruits and vegetables. [47]	
	About a third of participants said they did not consume more fruits and vegetables because they did not think about it. Several mentioned that they did not actively prioritize healthy eating behaviors, as illustrated by the following quote in response to the question about reasons for not eating more fruits and vegetables: “I don’t think about it.” [51]	
	Other noted perceived barriers to fruit and vegetable consumption included taste preferences: “Yeah, cauliflower is nasty.” [51]	
Lifestyle factors	““If [fruit and vegetables are] cut up or something and there, it’s ready to eat, I’ll eat it.” [52]	[37, 38, 43–46, 48, 49, 52]
	Although all key informants noted that it is easy to buy many different kinds of vegetables and fruits at the Asian grocery stores, some Hmong Americans are not eating as many vegetables and fruits because of lack of time and money to purchase the vegetables and fruits with which to prepare traditional meals with fresh ingredients. [45]	
	“Vegetables and fresh food is far dearer.” [48]	
Fast-food vs vegetables	“I do think emm to buy fresh fruit emm and fresh vegetables is a lot more expensive than them going to buy beans and chips.” [49]	[37, 49, 52]
	Students also said it is difficult to eat as recommended because taste is very important to them and "junk food" tastes better than more healthful options (e.g., fruits, vegetables, dairy products, and lower-fat products). [37]	
	They discussed cost as an issue and said that they like fast food because it is inexpensive, and they can get filled up for a few dollars, whereas eating salads or eating at another kind of restaurant with more healthful foods costs more. [37]	
Family, home & friends		
Home food environment	“The more children you have, the tighter your money is when it comes to buying groceries.” [45]	[37–39, 41, 43–46, 48, 51, 52]
	Only three students mentioned eating cooked vegetables during dinner at home. [39]	
	A majority of participants cited their home environment as an important source of healthy food, such as fruits and vegetables. [51]	
Parental influence	““I told her that this is how we always eat. She has to eat this to help her body, so that she doesn’t have diabetes and high blood pressure.” [45]	[38, 39, 41, 44, 45, 47, 48, 51]
	“I tell my child, ‘Eat vegetables so you’ll be strong,’ then he eats it. [45]	
	A participant described how food consumption at home was heavily influenced by parents’ choices and the availability of food items [51]	
	Several felt they ate healthier if adults at school and home provided healthy foods and encouraged their consumption [44]	
	Participants noted children ate more fruits and vegetables if their families were eating more fruits and vegetables. [38]	
	A major feedback structure identified was the normalization of more fruits and vegetables at home, which increased when the family ate more fruits and vegetables together, with adults role-modelling healthy eating. [38]	
External encouragement/support	Rather than purchasing fast food, families could also be encouraged to prepare healthy versions of fast foods, alternatively they could be encouraged to purchase a healthier meal option, e.g. charcoal chicken with a home-made salad. [52]	[45, 52]
Friends’ influence	Foods such as fruits and vegetables were not listed as items purchased in the presence of friends [47]	[47]
School		
Nutrition education	“Teachers at school talk about it. We watch some videos on vegetables and needing to exercise’’ [47]	[47]
Teachers’ support/encouragement	Several felt they ate healthier if adults at school and home provided healthy foods and encouraged their consumption [44]	[44]
School food environment	Many students said that they would eat fruits and vegetables if schools offered a variety and served them fresh. [40]	[37–42, 44–47, 51, 52]
	Students voiced awareness that fruits and vegetables were healthy but believed that current school food was “greasy” and unhealthy. [42]	
	Most participants said school lunch meals were a key source of healthy food and regularly offered fruits and vegetables in their community. [51]	
Community		
Local produce	Overwhelmingly, students across groups said that the best way to get students to care about eating local produce is to make it ‘‘tastes good’’ and ‘‘looks good.’’ [50]	[50]
Community nutrition knowledge & practices/norms	Participants identified the effect of community practices and norms on children’s fruit and vegetable intake, mentioning food prepared for community gatherings at church and on the marae (Maori meeting grounds) which could be either positive or negative in aiding fruit and vegetable intake. [38]	[38]
Local stores/restaurants	Most stated they would make healthier choices if “quality” restaurants and markets were available in their community. Examples offered included grocery stores that supply organic, fresh produce and natural foods. [39]	[38, 39, 41–45, 47, 51]
	In their description of corner stores and carry-outs, many indicated that fruits and vegetables are not readily accessible at these locations. Participants also indicated that when fruits or vegetables are available in nearby stores, the products are of poor quality and ’’nasty‘‘. [47]	
	A majority of participants perceived fruits and vegetables to be highly available in their community and said they could easily access these items at nearby grocery stores, their home, or their school. [51]	
Fast-food restaurants	Most participants ate only salads that included lettuce and tomatoes or had this added to a sandwich at school lunch or a fast-food restaurant meal. [39]	[37, 39]
	Participants stated that they seldom order salads or milk at a fast-food restaurant because they are not available or not promoted; they are not as visible as other options. Furthermore, if salad is available, they expressed concerns about its quality. [37]	

#### Individual level

##### Vegetables’ characteristics

Vegetables’ sensory attributes were identified as determinants of intake in several studies. Three studies conducted in the US included aspects such as smell [45], freshness [45], taste [37, 50], and appeal/appearance [37, 50] as intrinsic factors determining vegetable intake. Adolescents emphasized that the provision of fresh vegetables that smelled, tasted and looked good would encourage them to eat them [37, 50]. However, they noted that some vegetables, such as cauliflower, did not have a good taste *per se* [37]. In one study [45] conducted among Asian Americans, participants were concerned about the presence of chemicals in vegetables which, therefore, limited their vegetable intake.

##### Personal factors

Several individual factors were described as determinants of vegetable intake among adolescents from socioeconomically disadvantaged backgrounds. Considering knowledge about the health effects of vegetables, US participants’ deemed vegetables as healthy foods that should be often consumed as part of a healthy diet [41, 43, 45, 51]. Although the young people in the study from Northern Ireland [49] had very limited knowledge about concepts of healthy eating, they recognized vegetables as an important part of healthy eating. Participants were also aware of the effects that vegetables have on health in two US studies [41, 45], one of them carried out among a Hmong population [45]. In this study by Pham et al. [45], both adolescents and adults noted the importance of consuming vegetables to prevent future diseases such as obesity, diabetes and cardiovascular diseases. On the other hand, in another US study [43] conducted among Asian-American adolescents and parents, which also included Hmong people, participants had limited knowledge on the health benefits of eating vegetables. In addition, two studies found that adolescents’ knowledge about types of vegetables was limited as they could not distinguish between either fruits and vegetables [39] or different types of vegetables in a salad [45]. Lack of knowledge on the recommended intakes of vegetables was also described in the study targeting Asian-American participants [43]. Likewise, Hmong parents did not know the definition of a serving of vegetables, however, their adolescent children reported being familiar with vegetable servings [45]. Individual preferences and (dis)liking of vegetables also emerged as relevant determinants. In three studies, two from the US [39, 51] and one from Greece [48], young people reported a dislike for vegetables which limited their intakes, whereas other US adolescents described a liking for vegetables [44] and listed many types of vegetables they consumed [44, 45]. Adolescents’ preferences in general [51] or preferences for some specific types of foods, i.e., unhealthy foods, as opposed to vegetables [37, 41] or specific vegetables [45] had an influence on the amount of vegetables consumed. Preparation methods and adolescents’ preferences for some methods over others were also described to influence adolescents’ vegetable intake. Adolescents from both Australia [52] and the US [37] emphasized the need to cook or prepare vegetables in certain ways to make them more appealing. For instance, vegetables could be served with dip or with cheese sauce or could be stir-fried or hidden in a stew. In fact, hiding vegetables in other preparations such as in stews or in meatballs was a common technique that was suggested by both Australian adolescents [52] and Greek parents [48]. Two other psychosocial factors that emerged in only one study that applied the Social Cognitive Theory as framework were self-efficacy and outcome expectancies as adolescents expressed their willingness and that of their friends to eat healthy foods, including vegetables, and their motivation to eat vegetables to prevent suffering from obesity, diabetes and heart disease [47]. Lack of motivation or of interest were also reported as barriers to vegetable intake in two studies involving young adolescents from the US [51] and from Northern Ireland [49].

Other personal factors were reported in the studies. In the study by Pham et al. [45], adolescents said that they would eat more vegetables if they could do it while watching television or eating with friends. This reasoning was not further explored by the study researchers, and it is unclear why these two practices did not seem compatible with vegetable intake for these adolescents. Individual lifestyle factors reported to influence vegetable intake in studies in this review were price, convenience and time. Participants from the US [43, 45], Northern Ireland [49] and New Zealand [38] perceived vegetables as costly food items which, together with the lack of money experienced by these disadvantaged populations, represented a barrier to consumption. In addition to money-related issues, lack of time to buy and prepare vegetables [45], as well as their lack of convenience [37, 44, 52], also emerged as determinants of intake. Australian adolescents noted that if ready-to-eat vegetables were easily available and if they were easier and faster to prepare, they would eat them more often [52].

##### Vegetables versus fast food & other substances

Adolescents in five studies described their preferences for fast food as opposed to vegetables. One of the factors discussed was taste. US adolescents expressed how difficult it was for them to eat healthy foods, including vegetables, due to the fact that, for them, junk food tasted much better [37]. This same group of adolescents also discussed cost as an issue limiting their vegetable intake. They said that they preferred fast food because it was cheaper and they could satisfy hunger with less money than if they had to buy more healthful foods, such as salads, which they reported to be more expensive [37]. Similarly, both US [51] and Northern Irish [49] adolescents also perceived the cost barrier of vegetables as compared to apparently less costly items such as unhealthy foods [49, 51], or drugs [49]. In addition, two studies [38, 52] highlighted the phenomenon that fast food was displacing the intake of other healthier food items such as vegetables due to the availability of fast-food outlets in the community.

#### *Interpersonal level*

##### Family, home and friends

Adolescents’ vegetable intake seemed to be strongly influenced by their parents and the home food environment as described in several studies. Limited availability of vegetables in the household was mentioned by adolescents as a barrier to vegetable intake [37, 39, 45] and they said that having more vegetables at home would encourage them to eat them more frequently [45]. Hmong households with vegetable gardens reported eating a variety of vegetables [45], however they complained about not being able to grow as many varieties of vegetables as they did in their origin countries. In other studies, the home environment was described as an important source of healthy foods, including vegetables [38, 41, 43, 44, 51]. Family preparation methods also emerged as determinants of vegetable intake in adolescents. In three studies [43, 45, 48], vegetables were commonly included in meals as part of traditional cuisines, that is, in Asian and Mediterranean cultures. In another study carried out in Australia [52], adolescents pointed out that their parents should use more appealing cooking methods for vegetables beyond steaming and that families should be encouraged to prepare healthy versions of fast foods. In the study by Gerritsen et al. [38], participants noted that shared family meals, together with the family cooking skills and ability to prepare vegetables, were crucial to increase vegetable intake among adolescents. On the other hand, limited household budgets to buy groceries [45] and the availability of unhealthy competitive foods in the household [44, 52] were identified as barriers to vegetable intake in adolescents.

Several papers highlighted the major role of parents in influencing their children’s vegetable intake. In two studies, adolescents reported that their parents encouraged them to eat more vegetables [39, 44] and that although they initially protested, they ended up eating vegetables [39]. Likewise, adolescents in two studies [44, 45] pointed out that they would eat more vegetables if they were encouraged to do so by their parents, particularly their mothers. Parents in the study by Pham [45] also said that they often encouraged their children to eat vegetables given the benefits for their children’s health. Parental food choices [51] and parental role-modeling [38] were also identified as determinants of vegetable intake. Greek adolescents explained how simply observing their parents cooking vegetables made them eat those vegetables [48]. In two studies [45, 47], parents reported having rules and norms around vegetable consumption such as always having vegetables for dinner [47]. Furthermore, Gerritsen et al. [38] identified family barriers to vegetable intake that could potentially be caused by low-income employment including limited parental time to prepare vegetables and low household budgets which could lead them to prioritize satiety over healthy foods, such as vegetables, in their purchases. In two studies [45, 52], participants discussed the importance of providing families with more information on how to eat healthily and encouraging them to follow a healthy diet, including eating more vegetables.  Only one study [47] carried out in the US described the influence of friends on adolescents’ vegetable intake.  Adolescents in this study said that vegetables were not among those foods purchased when with their friends.

#### *Organizational level*

##### School

According to the studies included in the review, the school seemed to have a major influence in vegetable intake among adolescents from socioeconomically disadvantaged backgrounds. US adolescents pointed out that they ate healthier if adults at school provided healthy foods and encouraged them to eat these foods [44]. In addition, as described in another study [47], teachers were among those sources of information that provided nutrition education to US adolescents including videos on vegetables. The role of the school food environment was discussed in several studies. Limited school availability of vegetables was described in two studies [39, 45] and was considered as being one of the reasons why adolescents had low intakes [39]. On the other hand, adolescents in another study [51] reported to have high accessibility to vegetables at school, which was regarded as a key source of healthy foods. As mentioned by participants in five studies [37, 38, 42, 44, 45], they would eat vegetables if they were more available and accessible in their schools. In two studies [38, 44], it was suggested to provide vegetables for free or for sale at school to encourage their intake among young people. Freshness [40, 46, 51], variety [40, 44], appearance [37, 41, 42], smell [41], and taste [42] of school vegetables were identified as main determinants of intake. Other suggestions made by participants to increase adolescents’ vegetable intake were improving visibility of vegetables in the school canteen, providing braces-friendly vegetables and preparing vegetables in a more appealing manner [42]. The provision of more convenient and ready-to-eat vegetables was also suggested in one study [37]. Adolescents in another study [51] pointed out that the only sort of vegetable that they liked in the school canteen was the salads, but that they ran out quickly. Another determinant of vegetable intake was the availability of unhealthy competitive foods at schools [44]. In order to promote access to and availability of healthy foods in schools, including vegetables, Australian adolescents suggested to swap unhealthy foods at school for healthy foods [52].

#### *Community level*

##### Community

Adolescents’ vegetable intake was influenced by several characteristics of the community where they lived.  Greer et al. [50] investigated adolescents’ perceptions of local produce as a strategy to increase fruit and vegetables consumption. Adolescents showed very limited knowledge of the vegetables that could be grown locally. They suggested ideas to promote consumption of local produce in the school environment, which included showing adolescents that local produce tasted better and had a better appearance than other vegetables not produced locally. Besides, adolescents noted that freshness was a key feature of local produce that contributed to its taste and quality. The study by Gerritsen et al. [38] described how community practices and norms such as food prepared for community gatherings could either promote or discourage vegetable intake among community members, including young people. Participants highlighted the need to provide community nutrition knowledge together with growing and sharing healthy food including vegetables.

As reported in several studies [37–39, 41–45, 47, 51], the physical environment of the community such as local stores and restaurants, including fast-food restaurants, had a major impact on the adolescents’ intake of vegetables. The main barriers reported by participants were the lack of restaurants and markets selling vegetables in the community [39] together with limited accessibility to these places [47]. Vegetable availability and accessibility in local stores were also described in several papers. In some instances, participants said that vegetables were not accessible in these locations [41, 47] and that when they were available, their quality was very poor [42, 44, 47] and/or they were too expensive [42, 44]. Dodson et al. [41] noted that while fresh vegetables were available in local grocery stores, they were surrounded by sugary snacks. Besides, participants in this study described that street vendors of fresh vegetables were available in the community, but they were becoming scarce [41]. In one study [47], adolescents suggested that increasing the availability of vegetables in their local market would encourage them to eat them.  On the other hand, participants in three studies reported high availability of vegetables in their local communities [43, 45, 51], however, adolescents in the study by Payan et al. [51] noted that stores with higher quality healthy foods such as organic vegetables were not  available in the proximity of their homes.

Focusing on fast-food outlets, one study [37] reported that vegetables in these places were often not accessible or not promoted or visible. Besides, adolescents questioned the quality of these vegetables when they were visible. On the contrary, US adolescents in the study by Campbell [39] described fast-food meals as an opportunity to eat vegetables such as the lettuce and/or tomatoes that were added to these meals.

### Study quality assessment

The quality assessment of the studies is displayed in Table 3. There were 57 quality criteria items totaling to a maximum of 61 points, verbatim transcription and full publication of the interview guide counting double. Five studies [37, 42, 44, 51, 52] met ≥45 points, seven [38, 41, 46–50] studies met 39-44 points, and four [39, 40, 43, 45] studies met ≤34 points of the quality criteria. The minimum and maximum number of quality criteria met were 18 [40] and 49 [52], respectively.

**Table 3:** Quality assessment of included studies

**Author, year of publication**	**Campbell, 2009** [39]	**Chatterjee, 2016** [46]	**Christiansen, 2013** [47]	**Cullen, 2007** [40]	**Dalma, 2016** [48]	**Davison, 2015** [49]	**Dodson, 2009** [41]	**Gerritsen, 2019** [38]	**Goh, 2009** [42]	**Greer, 2018** [50]	**Harrison, 2005** [43]	**Kubik, 2005** [44]	**Neumark-Sztainer, 1999** [37]	**Payan, 2017** [51]	**Pham, 2007** [45]	**Stephens, 2015** [52]
**Aims**																
Aims and research questions are explicitly stated	Y	Y	Y		Y	Y	Y	Y	Y	Y		Y	Y	Y	Y	Y
Qualitative approach appropriate to answer research questions	Y	Y	Y	Y	Y	Y	Y	Y	Y	Y	Y	Y	Y	Y	Y	Y
**Preconceptions**																
Explicit theoretical framework or literature review and/or pre-study beliefs	Y		Y		Y	Y	Y			Y	Y	Y	Y	Y		Y
Information how theory is used (N/A if no theorical framework)		NA	Y	NA	NA	Y	Y	NA	NA	Y	NA				NA	Y
**Sampling procedure**																
Explicit sampling strategy of field sites and/or participants	Y	Y	Y	Y	Y	Y	Y	Y	Y	Y		Y	Y	Y	Y	Y
Recruitment strategy: how?		Y	Y	Y	Y	Y	Y	Y	Y	Y	Y	Y	Y	Y	Y	Y
Recruitment strategy: by whom?		Y			Y			Y	Y	Y		Y		Y	Y	Y
Explicit justification of sampling strategy		Y					Y	Y				Y	Y	Y		Y
Sampling strategy reflects the study purpose	Y	Y		Y	Y	Y	Y	Y	Y	Y	Y	Y	Y	Y	Y	
Sample size provided or can be estimated	Y	Y	Y		Y	Y	Y	Y	Y	Y	Y	Y	Y	Y	Y	Y
Non-participation described/response rate (N/A if voluntary sample)	NA				NA			NA			NA	NA	Y		NA	Y
Sampling/data collection continued until point of data saturation					Y				Y					Y		Y
**Ethical concerns: explicit statement about…**																
Informed consent (parent and/or adolescents)	Y	Y	Y	Y	Y	Y	Y	Y	Y	Y		Y	Y	Y		Y
Anonymity and confidentiality	Y															Y
Ethical approval/review	Y	Y	Y	Y	Y	Y	Y	Y	Y	Y		Y		Y	Y	Y
**Sample characteristics: Explicit and sufficient description of…**																
Gender of adolescent participants	Y	Y	Y			Y	Y	Y	Y	Y		Y	Y	Y	Y	Y
Age of adolescent participants or school year	Y	Y	Y	Y	Y	Y	Y	Y	Y		Y	Y	Y	Y	Y	Y
Race/ethnicity of the adolescent participants	Y	Y	Y	Y			Y	Y	Y	Y	Y	Y	Y	Y	Y	
**Data collection**																
Data collection method (e.g. focus groups, observations stated)	Y	Y	Y	Y	Y	Y	Y	Y	Y	Y	Y	Y	Y	Y	Y	Y
Explicit rationale for data collection method	Y		Y		Y	Y		Y				Y	Y	Y		Y
Data collection methods adequate to answer research question	Y	Y	Y	Y	Y	Y	Y	Y	Y	Y	Y	Y	Y	Y	Y	Y
Number of focus groups, interviews, observations provided		Y	Y	Y	Y	Y	Y	Y	Y	Y	Y	Y	Y	Y	Y	Y
Size of focus groups described or average can be estimated		Y	Y		Y	Y	Y	Y	Y	Y	Y	Y	Y	Y	Y	NA
Composition of adolescent focus groups/interviews described		Y				Y	Y	Y	Y			Y	Y			Y
Explicit rationale for focus groups/interview composition						Y		Y	Y							Y
Interview setting described	Y	Y	Y		Y	Y	Y	Y	Y	Y		Y	Y	Y		Y
Interviewer described (who? How many?)	Y	Y	Y		Y	Y		Y	Y	Y		Y	Y	Y		
Duration of interviews, focus groups, observations described	Y	Y	Y		Y	Y		Y	Y	Y		Y	Y	Y	Y	Y
**Interview guide**																
Interview guide used	Y	Y	Y	Y	Y	Y	Y	NA	Y	Y		Y	Y	Y	Y	Y
If yes: partly described (key questions)? Y, fully described? YY	YY		Y	Y	YY	Y	Y		YY	YY		Y	Y	YY		YY
**Analysis**																
***Reliability/consistency***																
Explicit information of audiotaping of interviews	Y	Y	Y		Y	Y	Y		Y	Y	Y	Y	Y	Y	Y	Y
Explicit information of transcription of interviews: Y, verbatim: YY	Y	YY	YY		Y	YY	Y		Y	YY	YY	YY	Y	YY	Y	YY
***Communicative validity***																
Analyst described (who? How many?)		Y	Y		Y	Y		Y	Y	Y		Y		Y		Y
Clear description of analytical method?		Y			Y	Y	Y	Y	Y			Y	Y	Y		Y
Explicit analytical approach (data-based or theory based)		Y	Y			Y		Y	Y	Y		Y	Y	Y	Y	Y
Analytical procedures appropriate to the research questions		Y	Y		Y	Y	Y	Y	Y	Y	Y	Y	Y	Y	Y	Y
Explicit rationale for choice of analytical procedures								Y					Y			
Sampling strategy/focus group composition is used in analysis											Y	Y	Y			
**Findings/presentation of findings**																
Clear presentation of findings	Y	Y	Y		Y	Y	Y	Y	Y	Y	Y	Y	Y	Y	Y	Y
Author's voices can always be distinguished from informant's voices	Y	Y	Y	Y	Y	Y	Y	Y	Y	Y		Y	Y	Y	Y	Y
Sufficient inclusion of quotes to support findings			Y		Y	Y	Y		Y			Y	Y	Y		Y
Clear description of selection and edition of quotes													Y			Y
Different participants' views can be distinguished		Y	Y			Y	Y			Y		Y	Y	Y		Y
The stated conclusion is supported by findings		Y	Y		Y	Y	Y	Y		Y	Y	Y	Y	Y	Y	Y
Relevance: Findings/conclusions illuminate the research questions	Y	Y	Y		Y	Y	Y	Y	Y	Y	Y	Y	Y	Y	Y	Y
**Internal validity**																
Description of validity and pilot-testing of applied instruments/guides			Y		Y					Y		Y	Y			Y
***Explicit strategies for validating presented findings***																
Researcher/analyst triangulation		Y	Y		Y	Y	Y	Y	Y	Y		Y	Y	Y		
Method triangulation	Y		Y	Y		Y	Y		Y		Y				Y	
Source triangulation		Y	Y	Y	Y	Y	Y	Y	Y		Y				Y	
Theory triangulation																
Peer debriefing/audit trail									Y							
Member checks/respondent validation							Y	Y								
Attention to negative or deviant cases																
**External validity**																
Discussion of transferability (applicability of findings in other contexts)			Y		Y		Y	Y	Y	Y		Y	Y	Y	Y	Y
Explicit reflections on selection bias/non-response of participants						Y	Y	Y					Y	Y	Y	Y
**Discussion**																
Adequate attention to previous knowledge and what the study adds	Y		Y		Y	Y	Y	Y	Y	Y		Y	Y	Y		Y
Findings provide new insight on potential determinants of vegetables	Y	Y	Y	Y	Y	Y	Y	Y	Y	Y	Y	Y	Y	Y	Y	Y
Discussions of limitations of qualitative study			Y			Y	Y	Y	Y	Y		Y	Y	Y	Y	Y
**Pragmatic validity**																
Discussion of implications for research and practice	Y	Y	Y				Y		Y	Y	Y	Y	Y	Y	Y	Y
**Total number of quality requirements met (out of 61)**	31	39	43	18	41	44	42	43	45	41	25	47	46	46	34	49

Y indicates ‘yes, information is provided in the manuscript’; a blank space indicates information about the criterion is not provided in the manuscript; N/A indicates that the criterion is not applicable or relevant for the study

Studies with low scores were characterized by insufficient description of the theoretical framework, and of the data collection and analysis methods, and lack of discussion of study limitations, of transferability of findings and of the contribution of study findings to previous research. Further, low scores were found when there was a lack of data validation strategies. For one of these studies [40], the qualitative element was a secondary aim and, therefore, a detailed description on how it was conducted was not provided.

## Discussion

Multiple determinants of vegetable intake among adolescents from socioeconomically disadvantaged backgrounds were identified. Furthermore, the review provided additional determinants of vegetable intake within this population group that have scarcely been investigated in quantitative studies and that were not identified in our previous review of determinants investigated through studies of a quantitative nature [29]. These newly identified determinants were: sensory attributes of vegetables beyond taste such as smell, appeal/appearance and freshness including those of local produce and of vegetables in school and local stores; cost and lack of convenience of vegetables and time-consuming preparation and cooking methods; lack of motivation, interest and prioritization to eat vegetables; preference for fast food due to better taste, lower prices, and better satiating attributes, and availability of unhealthy competitive foods in the home and school environment; household-related aspects such as parental preparation methods, cooking skills and cultural factors, household budget and prioritization of satiety over nutrition, parental time, outcome expectancies and nutrition knowledge, and shared family meals; schoolteachers’ encouragement to eat vegetables and school vegetable availability in terms of amount, variety, visibility, convenient and ready-to-eat vegetables and use of more appealing preparation methods; local produce awareness; community nutrition knowledge and practices around vegetable intake; and accessibility to and availability of vegetables in fast food restaurants.

Unsurprisingly, vegetables taste seems to strongly influence vegetable intake among young people. Given the genetic predisposition of human beings to reject those foods that are bitter or sour [53], it is reasonable to think that individuals, particularly young populations, tend to prefer other foods over vegetables. Besides, some vegetables such as broccoli or cauliflower have a very characteristic taste that may discourage young people to eat them. In agreement with Krølner et al. [8], other sensory attributes such as smell, freshness and appeal/appearance of vegetables were identified as determinants of intake. Taste, smell and textures responses are influenced by a range of genetic, physiological, and metabolic variables [54]. Although it is known that sensory responses alone do not predict food consumption, they do shape food preferences and eating habits [54]. In fact, individual preferences and (dis)like for vegetables emerged as critical determinants of vegetable intake among adolescents from socioeconomically disadvantaged backgrounds. Early and continuous exposure to vegetables may be crucial to overcome vegetables aversion among children and adolescents [55–57]. Furthermore, using cooking methods that make vegetables more appealing or adding other ingredients such as herbs or spices [58] may also help to increase vegetable acceptance in young populations. In this regard, our findings showed adolescents’ preference for certain preparation methods to make vegetables more appetising. Another aspect that was identified as a barrier to vegetable intake among adolescents from socioeconomically disadvantaged backgrounds was their preference for other unhealthy foods such as fast food as opposed to vegetables. These foods are characterized by being rich in fat, sugar and salt which makes them more palatable and tastier than vegetables. This perception of vegetables being less tasty than other foods may be explained by earlier experience or lack of experience with vegetables [59]. As food choices are mainly determined by the perceived tastiness of food products, providing vegetable tasting opportunities to young people from socioeconomically disadvantaged backgrounds at an early age may have a positive effect on their acceptance and may encourage their selection among this population group [59–61].

Findings on vegetable knowledge were inconsistent. In some studies, participants reported being aware of the importance of eating vegetables as part of a healthy diet as well as of the benefits of vegetable consumption on health; however, young people in other studies showed poor ability to distinguish between types of vegetables or between fruit and vegetables. Furthermore, parents did not know the recommended intakes of vegetables or the definition of a serving of vegetables. Knowing about vegetables and their properties can be a first step to encourage individuals to improve their consumption. However, previous reviews focusing on quantitative studies [7, 62, 63] have reported mixed findings about the association between nutrition knowledge and fruit and vegetable intake in young populations. In our review of quantitative determinants of vegetable intake among adolescents from socioeconomically disadvantaged backgrounds, we failed to observe a consistent association between knowledge about vegetables and their intake [29]. According to the Social Cognitive Theory [64], having the knowledge about a specific behavior is not sufficient for this behavior to take place. Aspects such as self-efficacy, outcome expectations and motivation are also critical. Self-efficacy, which is defined as the individual’s belief on their capacity to successfully execute a behavior to attain a particular outcome [65], needs to co-occur together with having positive expectancies of that outcome [64] in order to engage individuals in a specific behavior. Unexpectedly, and despite their relevance, these two psychosocial factors only emerged as determinants of vegetable intake in one study. According to Bandura [64], in addition to self-efficacy and outcome expectancies, the goal also needs to be valued by the participants. In this sense, lack of motivation and interest to eat vegetables to be healthy also emerged as barriers to vegetable intake among adolescents from socioeconomically disadvantaged backgrounds. Often, these populations are exposed to many living difficulties at home and within their communities that eating healthy may be the least of their priorities. However, if individuals are not able to value and prioritize the intake of healthy foods and of vegetables, it will be extremely difficult to encourage their intake.

Vegetables were frequently regarded as costly products, even across different countries, representing a huge barrier for families with low household budgets. For that reason, adolescents and their families tended to prioritize other more affordable and satiating foods, including unhealthy foods, over vegetables. This could partially explain why recent evidence shows that adolescents from poorer backgrounds have significantly lower intakes of fruit and vegetables than those from more affluent ones [3]. Furthermore, the perceived higher cost of vegetables could also lead adolescents from socioeconomically disadvantaged backgrounds to prioritize other habits over consuming vegetables. Therefore, providing more affordable vegetables could encourage heathier food choices within this population group. In addition to cost, vegetables’ lack of convenience and lack of time among adolescents and families were also considered as crucial determinants of their intake. This lack of time among families, due in part to low-income jobs and long working hours, seemed to lead families to consume more convenient foods that can be prepared quickly. Although it may be difficult to overcome this barrier, families should be supported and enabled to eat healthy within their budget. Future intervention programs should aim to improve cooking skills as they may be limited within families from socioeconomically disadvantaged backgrounds. [38]. At the more global level, both the food industry and state governments also have a role in supporting these families to eat more vegetables. Among other initiatives, the food industry could make more efforts to provide easy-to-use or -eat vegetables and/or to reduce the price of vegetables by means of offers or discounts [66]. On the other hand, governments could provide vegetable growers and producers with subsidies to scale up domestic horticulture production which would make vegetables more accessible and affordable to families while helping address climate change.

Parents and the household food environment were also identified as major determinants of vegetable intake among adolescents. As reported in several systematic reviews, there is extensive research of a quantitative [7, 62, 63, 67, 68] and of a qualitative nature [8] on how parental behaviors, attitudes and knowledge, together with the food available at home, influence their children’s food intake, including vegetables, regardless of their socioeconomic level. As described in two studies [45, 52], families from socioeconomically disadvantaged backgrounds should be provided with external support to help them adopt healthy diets and consume more vegetables. In this sense, free activities including nutrition education, food tasting or practical cooking sessions, among others, could be offered at the community level. Findings from two intervention studies carried out among low-income adults reported a significant increase in fruit and vegetable intake [69] and an improvement in dietary behaviors, including dietary quality [70], after attending community-based nutrition education programs. However, it is important to note how the impact of the home environment can lessen as adolescents get older. In the Health Behaviour in School-aged Children survey, daily intake of vegetables was lower in older adolescents in almost half of the countries/regions included in the report. Adolescents gain more autonomy over their eating behavior while growing up and are more likely to make unhealthy choices and skip meals [3]. This occurs in parallel with the fact that parental influence gradually shifts to peers’ influence as adolescence progresses [71]. As reported in the review by Krølner et al. [8], peers influence on adolescents’ food choices does not seem to support vegetable intake due to the strong peer pressure to eat unhealthy food. Unexpectedly, the influence of friends on vegetable intake was only described in one of the studies included in this review.

It is well known that the school food environment exerts a strong influence on adolescents’ dietary behaviors [72]. Availability of foods with poor nutritional quality in the school hinders the acquisition of healthy eating habits among adolescents [73]. Adolescents attending socioeconomically disadvantaged schools have been shown to be less likely to consume vegetables on a daily basis [74]. Vegetable availability and accessibility in school were identified as crucial determinants of vegetable intake; however, as already identified in the review by Krølner et al. [8], other aspects such as freshness, amount and variety, appearance, smell, and taste were regarded as important by the adolescents to encourage vegetable intake in the school setting. Increasing vegetables visibility, using preparation methods to make vegetables more appealing and providing more convenient options could promote vegetable intake in these settings. Improving the provision of healthy food in schools by considering aspects such as food aesthetics and freshness, among others, has been suggested to be effective in improving dietary habits in adolescents from socioeconomically disadvantaged backgrounds [73]. Nevertheless, it seems that the most promising approach to increase vegetable intake among these adolescents is to offer complimentary vegetables and school lunches [73]. However, the huge availability of fast foods not only in the school premises but also near the schools may jeopardize the potential benefits that school initiatives on healthy eating may have on the dietary habits of adolescents. Previous research has shown an inverse association between adolescents’ vegetable intake and the presence of fast-food environments around secondary schools [75]. For that reason, although schools can have a major role in promoting healthier dietary habits and in reducing health inequalities, a whole systems approach to school policy by combining environmental and behavior change together with food and nutrition education is needed from a regional and/or national perspective. This would guarantee the adoption and effectiveness of healthy eating strategies in socioeconomically disadvantaged areas [73].

The food environment of the neighborhood, which includes a mixture of retail outlets, restaurants and take-away (fast-food) outlets, influences individual food choices and food intake through the concept of food access [76, 77]. Food access is defined by five dimensions: availability, accessibility, affordability, accommodation and acceptability [78]. The main barrier to vegetable purchase in the urban community identified in this review was the lack of access to retail outlets such as markets and restaurants selling vegetables. In agreement with Krølner et al. [8], when vegetables were available in local stores, they tended to be low quality and/or have high prices. Therefore, at least four out of the five dimensions that should ease food access, particularly vegetables access, seem to be unmet in deprived areas which may limit vegetable purchase among those residing in these areas. This is coupled with the significantly higher fast-food outlet density that it is being observed in recent decades in more deprived areas [79, 80]. Even though easy access to these sorts of outlets has been shown to increase fast-food intake [81], vegetables such as lettuce, tomatoes, cucumber, etc., could also be incorporated in their menus so that they could be added to some of the fast-food meals. As described in the studies included in this review, good quality vegetables would need to be made more visible and accessible in these premises in order to encourage their intake among frequent fast-food consumers. Promoting local produce could represent another potential solution to increase vegetable intake in deprived areas as it could provide access to a variety of affordable and good quality vegetables.

The use of a theoretical framework was not common among the majority of the studies included in this review. The use of a theory in qualitative research provides a guide or framework for the study and justifies the methodological choices, among other aspects [82]. A strong theoretical framework can assist researchers in data coding and interpretation and can allow the identification of existing predispositions about the study [82]. However, excessive dependence on theories can hinder the importance of the data from coming through and researchers are asked to use them in a balanced manner. Nevertheless, it is recommended to incorporate theoretical frameworks in the construct and design of qualitative work to enhance the explanatory power and legitimacy of qualitative research [82].

### Strengths and limitations

Previous research has noted that exhaustive literature searches of qualitative research may be limited due to inconsistent indexing and use of search terms in databases together with the lack of specific databases exclusively devoted to qualitative health research [8, 83–85]. The fact that only 16 studies were included in the review can be explained by the fact that (1) research on this topic targeting adolescents from socioeconomically disadvantaged backgrounds is relatively scarce, and/or that (2) qualitative studies that were not indexed correctly by our search terms were excluded. We tried to mitigate this issue by screening the reference lists of previously published reviews and of the studies included in this review. Furthermore, studies identified through the literature search for quantitative studies were also included. This resulted in eight additional studies from which only one met the inclusion criteria. Another limitation is the high subjectivity associated with the analyses and interpretation of qualitative data; therefore, it cannot be ruled out that a different team of researchers would have interpreted the data differently. Furthermore, in three studies [43, 48, 49], the study settings were not explicitly described as either urban or rural; therefore, we cannot preclude that some of the data included in this review were obtained from rural communities.

One strength of this review is that we included papers published in five different languages as understood by the authors; however, we only found eligible studies published in English. As noted by Krølner et al. [8], it may be difficult to translate quotations into English and to report and interpret the findings in a different language of that used in the study. Indeed, among the 16 studies included in this review, only one was conducted in a non-native English-speaking country, i.e., Greece. Another strength is the fact that we did not only include the findings about the adolescents’ own views and perceptions, but also those from their parents, schoolteachers, youth workers, etc., when available. Triangulation of sources is considered as a strategy to test the validity of the data through the convergence of information from different data sources [86]. Furthermore, we applied systematic and standardized procedures to review and evaluate the papers included in the review, which can be considered as another strength.

## Conclusion and recommendations

To the best of our knowledge, this is the first systematic review that synthesized qualitative literature exploring the factors influencing vegetable intake among adolescents from socioeconomically disadvantaged backgrounds. This review identified multiple determinants of vegetable intake complementing those investigated in quantitative studies. These factors include sensory attributes of vegetables, psychosocial factors, lifestyle factors, fast food properties as opposed to vegetables, home food environment and parental influence, friends’ influence, school food environment, nutrition education and teachers’ support, availability and accessibility of vegetables in the community and community nutrition practices. Future large scale quantitative studies should attempt to examine the relative importance of these determinants in order to guide the development of successful interventions in this population group.

It should be noted that some of the determinants described in this review, such as vegetables’ sensory attributes or adolescents’ food preferences, among others, have also been reported among adolescents in general, regardless of their socioeconomic background [8, 63]. However, this review has contributed to identify several determinants that are specific to adolescents from more deprived backgrounds and that could explain the low intakes of vegetables within this population group. For example, these adolescents, in particular, seem to lack motivation and interest to eat vegetables which is coupled with the fact that they consider vegetables as costly, inconvenient and poorly satiating food items in comparison with other unhealthy foods. In addition, household financial resources are limited most of the times, parents do not have enough time to cook, and they are often forced to prioritize convenience and satiety over nutrition. Also, foods with poor nutritional quality are common in schools and vegetables are scarcely available and/or of low quality. Furthermore, these adolescents usually live in neighborhoods where fast-food outlets are extensively available whereas vegetables availability is frequently low or non-existent. In addition, access to and availability of vegetables in local stores other than fast-food outlets is limited, and the quality of the products, when available, tends to be poor. Keeping family, school and community factors in mind, future research should find out the reasons why adolescents in this population group in particular lack interest and motivation to eat healthy. Intervention programs should aim to change the preconceptions that adolescents have about vegetables by showing them and their parents that these products can be inexpensive, can be prepared relatively quickly and in a satiating and healthy manner.

One of the conclusions that can be drawn from this review through the application of the SEM to report the findings is the lack of evidence at the public policy level. Therefore, there is a need to develop public policies and actions that support families in more socioeconomically disadvantaged circumstances to eat healthier and to increase their intake of vegetables, mainly among adolescents. At the community level, policies should look at facilitating access to and availability of vegetables in socioeconomically deprived neighborhoods by supporting and promoting local produce and local stores. Furthermore, certain vegetables may need to be subsidized to make them more competitive against other unhealthy food items and families can purchase these products at lower prices. In addition, specific policies may target fast-food outlets to include a wider range of vegetables in their menus. While policies to promote healthy eating in schools are widely available across countries, in many instances there is no strong system in place to implement those policies. Therefore, governments need to invest more to support healthy food environments in schools.

Overall, this review has provided new insights on the determinants of vegetable intake among adolescents from socioeconomically disadvantaged backgrounds that are not easily captured through quantitative research. It has also shown how qualitative and quantitative research complement each other by providing a more comprehensive overview of the specific aspect under study. 

## Supplementary Information

**Additional file 1.**

**Additional file 2.**

## Data Availability

All the extracted data are available from the published peer-reviewed papers which are cited in the reference section.

## Abbreviations

WHO: World Health Organization
HBSC: Health Behaviour in School-aged Children
US: United States
SEM: socio-ecological model
