# Supplementary material for: What are the determinants of vegetable intake among adolescents from socioeconomically disadvantaged urban areas? A systematic review of qualitative studies
Source: Int J Behav Nutr Phys Act. 2022 Dec 26;19:158. doi: 10.1186/s12966-022-01396-9 (PMC9793665; doi:10.1186/s12966-022-01396-9)
Supplement: Supplementary file 2 — Additional file 2. [file 12966_2022_1396_MOESM2_ESM.docx]

**Additional file 1.** Search strategy and search terms used in PubMed.

| #1 Vegetable | vegetable* [MeSH] OR vegetable*[Title/Abstract] |
| --- | --- |
| #2 Population | adolescen*[MeSH] OR adolescen*[Title/Abstract] OR teen*[Title/Abstract] OR youth*[Title/Abstract] |
| #3 Qualitative methods and methodologies | anthropology[Title/Abstract] OR anthropologic*[Title/Abstract] OR ethnography[Title/Abstract] OR ethnographic*[Title/Abstract] OR qualitative[Title/Abstract] OR focus group*[Title/Abstract] OR interview*[Title/Abstract] |
| #4 = #1 AND #2 AND #3 | (((vegetable* [MeSH] OR vegetable*[Title/Abstract])) AND (adolescen*[MeSH] OR adolescen*[Title/Abstract] OR teen*[Title/Abstract] OR youth*[Title/Abstract])) AND (anthropology[Title/Abstract] OR anthropologic*[Title/Abstract] OR ethnography[Title/Abstract] OR ethnographic*[Title/Abstract] OR qualitative[Title/Abstract] OR focus group*[Title/Abstract] OR interview*[Title/Abstract]) |
| #5 | Limit #4 to humans and English, Spanish, Catalan, French and Portuguese |

**Additional file 2**

**Table.** Summary table of findings, codes and themes (an example).

| **Study** | **Text extracts** | **Quotes** | **Codes** | **Sub-themes** | **Themes** |  |
| --- | --- | --- | --- | --- | --- | --- |
| **Campbell, 2009 US** | Most participants ate only salads that included lettuce and tomatoes or had this added to a sandwich at school lunch or a fast-food restaurant meal. |  | Liking/preferences of limited vegetables | Cognitive factors | Personal factors |  |
|  | Reasons for not eating vegetables included dislike of cooked vegetables or unavailability at home, and limited availability at school lunch. |  | Dislike of vegetables | Cognitive factors | Personal factors |  |
|  | Participants’ knowledge of food groups was minimal. Several did not distinguish between fruits and vegetables. |  | No distinction fruits and vegetables | Nutrition knowledge/Cognitive factors | Personal factors |  |
| **Chatterjee, 2016**  **US** | Specifically, families noted cooking the same, healthy, foods—broccoli or baked chicken, for example—at home in a way that was more satisfying to children than what is prepared at school. |  | Preparation method |  | Personal factors |  |
| **Davison, 2015 Northern Ireland, UK** | For the young people concepts of healthy eating were limited and largely associated with consumption of fruit and vegetables. | ‘Eating fruit and veg each day’ (FG1, P2) ‘Like fruit and vegetables, fresh’ (FG2, P3) ‘Like your meant to have like 1/3 vegetables’ (FG3, P3) | Limited knowledge about nutrition, vegetables as healthy food | Nutrition knowledge/Cognitive factors | Personal factors |  |
|  | The notion that healthy food cost more than junk food was echoed by the service providers. | ‘I do think emm to buy fresh fruit emm and fresh vegetables is a lot more expensive than them going to buy beans and chips’ (PInt 6) | Vegetables as expensive food | Lifestyle factors | Personal factors |  |
|  |  |  | Lack of interest/no vegetables prioritisation | Cognitive factors | Personal factors |  |
| **Dodson, 2009**  **US** | Vegetables (e.g., broccoli), and fruits (e.g., apples, oranges) are classified as “clean” or “home foods”. Vegetables are described as “how we grow”, “giving you minerals, calcium, and calories” while broccoli can “help you be healthy”. Vegetables, fruits, and water were identified as healthy foods, while greasy and sugary foods were identified as unhealthy. |  | Knowledge about vegetables properties | Nutrition knowledge/Cognitive factors | Personal factors |  |
|  | However, teens rarely chose to eat these healthy foods over the unhealthy foods. |  | Preferences for some foods over others | Cognitive factors | Personal factors |  |
| **Kubik, 2005 US** |  | “Everything is convenience nowadays and who wants to like sit there and make an elaborate salad when you can just run to Taco Bell and get your fix, you know?” | (lack of) Convenience | Lifestyle factors | Personal factors |  |
|  | Students reported liking fruits and vegetables, providing long lists of their favorites. |  | Vegetables liking | Cognitive factors | Personal factors |  |
| **Payan, 2017 US** | When asked to describe a healthy meal, the most popular answer was “fruits and vegetables” in every group. |  | Nutrition knowledge about vegetables | Nutrition knowledge/Cognitive factors | Personal factors |  |
|  | Participants said they did not like eating vegetables |  | Liking | Cognitive factors | Personal factors |  |
|  | Other noted perceived barriers to fruit and vegetable consumption included taste preferences | “Yeah, cauliflower is nasty.” | Taste preferences | Cognitive factors | Personal factors |  |
|  |  | “Maybe like, because my Mum cooks really good things which are healthy at home, that are pretty delicious, so ask the kids like, ‘What do you want?’ instead of just making them steamed vegetables and all that, so you can have something else which is still healthy.” | Preparation methods |  | Personal factors |  |
| **Stephens, 2015 Australia** |  | “If food was more easier to prepare. I already know that [people] have the jars with the tomato sauce and all they have to do is put it in the pan with the meat and the pasta and that's pretty easy. So maybe if that was advertised more, like quick meals and stuff.” | Convenience | Lifestyle factors | Personal factors |  |
|  |  | “If [fruit and vegetables are] cut up or something and there, it’s ready to eat, I’ll eat it. If there was like less junk food and like only a few stuff of like chip bags and stuff.” | Convenience | Lifestyle factors | Personal factors |  |
|  |  | "Fruits and vegetables are harder to fix sometimes and take more time" | Convenience & time | Lifestyle factors | Personal factors |  |
| **Neumark-Sztainer, 1999 US** | Some said that fruits, vegetables, and dairy foods are not as convenient as other food because fruits have to be peeled, vegetables need to be cooked, and it is hard to get fruits or vegetables at a fast-food restaurant. |  | Lack of convenience | Lifestyle factors | Personal factors |  |
